# Assessing and testing anomaly detection for finding prostate cancer in spatially registered multi-parametric MRI

**DOI:** 10.3389/fonc.2022.1033323

**Published:** 2023-01-05

**Authors:** Rulon Mayer, Baris Turkbey, Peter Choyke, Charles B. Simone

**Affiliations:** ^1^ Department of Radiation Oncology, Perelman School of Medicine, University of Pennsylvania, Philadelphia, PA, United States; ^2^ OncoScore, Garrett Park, MD, United States; ^3^ Molecular Imaging Branch, National Institutes of Health (NIH), Bethesda, MD, United States; ^4^ Department of Radiation Oncology, New York Proton Center, New York, NY, United States; ^5^ Memorial Sloan Kettering Cancer Center, New York, NY, United States

**Keywords:** anomaly and outlier detection, multi-parametric MRI, prostate cancer, tumor detection, regularization, principal component analysis, image analysis, color analysis

## Abstract

**Background:**

Evaluating and displaying prostate cancer through non-invasive imagery such as Multi-Parametric MRI (MP-MRI) bolsters management of patients. Recent research quantitatively applied supervised target algorithms using vectoral tumor signatures to spatially registered T1, T2, Diffusion, and Dynamic Contrast Enhancement images. This is the first study to apply the Reed-Xiaoli (RX) multi-spectral anomaly detector (unsupervised target detector) to prostate cancer, which searches for voxels that depart from the background normal tissue, and detects aberrant voxels, presumably tumors.

**Methods:**

MP-MRI (T1, T2, diffusion, dynamic contrast-enhanced images, or seven components) were prospectively collected from 26 patients and then resized, translated, and stitched to form spatially registered multi-parametric cubes. The covariance matrix (CM) and mean μ were computed from background normal tissue. For RX, noise was reduced for the CM by filtering out principal components (PC), regularization, and elliptical envelope minimization. The RX images were compared to images derived from the threshold Adaptive Cosine Estimator (ACE) and quantitative color analysis. Receiver Operator Characteristic (ROC) curves were used for RX and reference images. To quantitatively assess algorithm performance, the Area Under the Curve (AUC) and the Youden Index (YI) points for the ROC curves were computed.

**Results:**

The patient average for the AUC and [YI] from ROC curves for RX from filtering 3 and 4 PC was 0.734[0.706] and 0.727[0.703], respectively, relative to the ACE images. The AUC[YI] for RX from modified Regularization was 0.638[0.639], Regularization 0.716[0.690], elliptical envelope minimization 0.544[0.597], and unprocessed CM 0.581[0.608] using the ACE images as Reference Image. The AUC[YI] for RX from filtering 3 and 4 PC was 0.742[0.711] and 0.740[0.708], respectively, relative to the quantitative color images. The AUC[YI] for RX from modified Regularization was 0.643[0.648], Regularization 0.722[0.695], elliptical envelope minimization 0.508[0.605], and unprocessed CM 0.569[0.615] using the color images as Reference Image. All standard errors were less than 0.020.

**Conclusions:**

This first study of spatially registered MP-MRI applied anomaly detection using RX, an unsupervised target detection algorithm for prostate cancer. For RX, filtering out PC and applying Regularization achieved higher AUC and YI using ACE and color images as references than unprocessed CM, modified Regularization, and elliptical envelope minimization.

## Introduction

Optimal prostate cancer (PCa) management requires an accurate evaluation of potential tumor aggressiveness (1–3). Several clinical indicators (4–17), such as prostate specific antigen (PSA) (7–9), seminal vesicle involvement (10, 11), tumor volume (12–16), extraprostatic extension and other MRI features (17–21), and the Gleason score (22) predict clinical outcomes such as biochemical recurrence after treatment (4–6) and cancer metastasis (10, 11, 14). However, some data, such as PSA (8), are not consistently predictive of outcome. By interpreting multi-parametric MRI using the Prostate Imaging Reporting and Data System (PI-RADS) protocol (17–20), radiologists apply a series of rules to generate a PI-RADS score for the lesion. Visual inspection and assessment of imaging or histology slices rely on the experience and judgment of radiologists and pathologists (17–20), possibly creating inconsistent evaluations due to inter-reader variability. A more objective and quantitative approach could reduce such variability.

To achieve this, there is interest in quantitatively applying and assessing supervised target algorithms to spatially registered T1, T2, diffusion, and dynamic contrast enhancement images at the voxel level (23–28). Previous research examined tumors using vectoral tumor signatures that were inserted into supervised target algorithms applied to spatially registered MRI hypercubes. The supervised target detection algorithms applied to (23–28) assessing prostate cancer were adapted from remote sensing applications designed to analyze hyperspectral images generated from airborne platforms. The spectral signature helps discriminate tumors (or targets in the case of remote sensing) from normal tissue (or backgrounds). Instead of exploiting the unique spectral content of a target, remote sensing can also peruse an image for candidate targets by examining and finding pixels (or voxels) that statistically depart from the background, also known as anomaly detection. Although less specific than supervised target detection, anomaly detection (29–31) can find unsuspected targets but is also subject to detecting additional false positives. For multispectral or hyperspectral images, the commonly used algorithm is called Reed-Xiaoli (RX) (32) and computes the magnitude of a voxel’s vector distance (in whitened space) from the background or normal prostate. The larger the RX value, the more the voxel departs from normal tissue. A hypersphere decision surface surrounding the background provides a criterion for whether a voxel is normal (inside the hypersphere) or anomalous (outside the hypersphere).

Anomaly detection has also been applied to medical images (33–38). However, this study significantly differs from previous efforts. The background (normal prostate) is characterized by second-order statistics such as a multi-dimensional covariance matrix and mean, not features derived through spatial processing. Specifically, most of the other studies extracted features from images derived in a single modality, unlike the work described in this manuscript. Previously, extra dimensions were added (37, 38) through spatial processing from a single modality. In another study, anomaly detection followed the temporal evolution (35) of a contrast agent from a single modality and considered time to be the “fourth dimension.” In this study, voxels from structural (T1, T2), dynamic contrast enhancement, and diffusion images composed the “fourth dimension.” The RX algorithm is purely spectral and does not require spatial processing. In addition, other studies employed deep learning and artificial intelligence approaches, unlike the present work, which used a faster, simpler algorithm (RX). The present work, unlike deep learning approaches that require retraining, can more easily be adapted to clinics that employ different magnetic fields and pulse sequences (23) by using whitening –dewhitening transforms.

This study of spatially registered multi-parametric MRI is the first to apply an unsupervised target detection for prostate cancer, specifically the RX algorithm, that does not use a tumor signature. The covariance matrix (CM) and mean background vector proscribes the decision surface for RX, an anomaly detector. The anomaly detector searches for voxels that depart from the normal tissue, or background. Such an anomaly detector may be sensitive to both higher and lesser vascularized regions of the tumor and provide a more complete assessment of the lesion.

## Method and materials

### Overall summary

**Figure 1:**
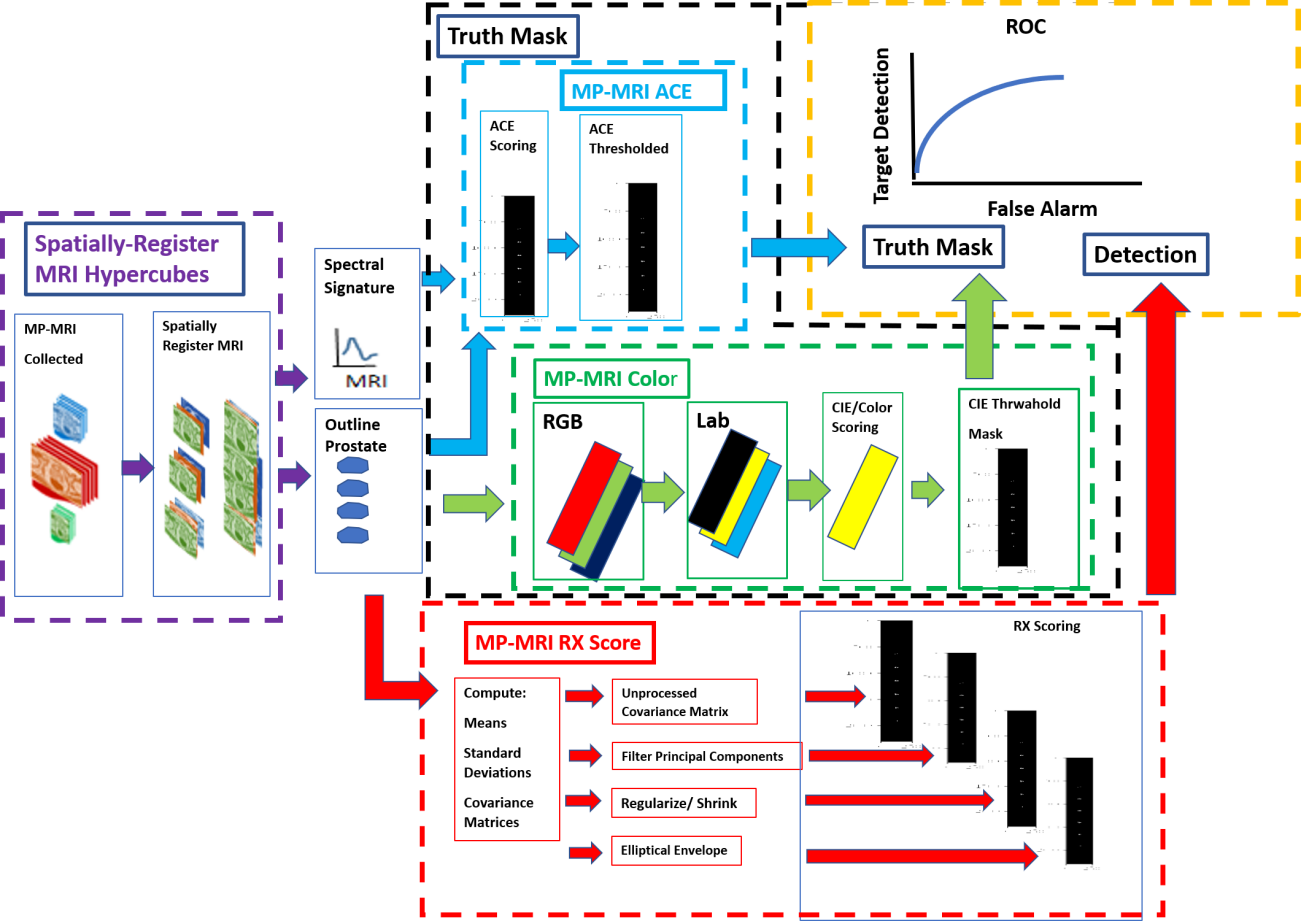
Schematic overview of processes that need to generate ROC curve (yellow outline). A spatially registered hypercube (purple outline, arrow) is composed of MRI modalities. Reference mask options include ACE (blue outline) and CIELAB (green outline). Detection map from the RX computation (red outline, arrow).

**Figure 1** shows schematically the procedures for computing anomaly detection and generating the receiver operator characteristic (ROC) curve. First, the MRI images are collected, resized, translated, resampled, and stitched together to assemble spatially registered hyperspectral image cubes. The normal prostate is outlined to help form a mask that is applied to the hyperspectral image cubes to aid calculations for RX (red outline, arrow) and Adaptive Cosine Estimator (31) or ACE (blue outline, arrow), a supervised target detection algorithm. The normal prostate mask aids the hyperspectral statistics computation for the normal prostate and delineates the normal prostate reference mask (orange arrow) for the ROC calculations. The color (green outline) scheme, specifically the RGB (red, green, and blue channels), is assigned to the washout from the dynamic contrast enhancement and the high B from the diffusion weighted images, respectively. The colors are quantified using CIELAB computations (24). Color/CIELAB is used to display tumors, to generate a Reference Mask. Thresholds applied to the ACE (blue outline) and CIELAB color (green outline, arrow) calculations help form two independent tumor reference image masks that are used for the ROC calculation (yellow outline). A signature of the tumor is taken from the hyperspectral image cube and inserted into the ACE calculation. Four options were examined for reducing noise in the covariance matrix for the RX calculation (red outline): principal component filtering, regularization, modified regularization, and the unprocessed covariance matrix (red outline).

The *Methods and materials* section qualitatively describes the individual components, namely the spatial registered hypercube assembly, reference mask, anomaly detector generator, and assessment. The Appendix summarizes the mathematics used to generate the components. More details can be found in the cited references.

### Spatial registered hypercube assembly: Study cohort

The Cancer Imaging Archive (TCIA) (39, 40), affiliated with the National Institutes of Health (NIH), collected and stored patient data from prostate tumor MRI and histology from whole-mount prostatectomy specimens. This study followed the Declaration of Helsinki (as revised in 2013). Since the images were anonymized, this investigation was determined to be IRB-exempt. This study followed the Health Insurance Portability and Accountability Act guidelines. A total of 26 patients were included. All patients had biopsy-proven adenocarcinoma of the prostate, with a median patient age of 60 years (range, 49 to 75 years), a median PSA of 5.8 ng/ml (range, 2.3 to 23.7 ng/ml), and a median GS of 7 (range, 6 to 9). Eighteen of the 26 patients had tumors larger than 1 cc. This study placed no restrictions on tumor location within the prostate. Robotic-assisted radical prostatectomy was performed at a median time of 60 days (minimum 3 days, maximum 180 days) following MRI without any intervening treatment.

### Spatial registered hypercube assembly: Whole mount prostatectomy and histology

The whole mount prostatectomy histology has previously been described in detail and is very briefly summarized (41–43). Following radical prostatectomy, the specimen was fixed at room temperature in formalin for 2 to 24 h and then placed in a customized 3D mold that is based on MRI and sliced in sections with a separation of 6 mm in the axial direction, corresponding to the MRI axial plane section. The individual tumor foci, dimensions, and Gleason scores from the histology slides were independently determined by two experienced pathologists who were blinded to the MRI results.

### Spatial registered hypercube assembly: Magnetic resonance imaging

The MRI collection was composed of structural (T1, T2) images, diffusion-weighted images (DWIs), and dynamic contrast-enhanced (DCE) images. The pulse sequences were described in earlier studies (41–43). This MRI protocol included triplanar T2W turbo spin echo, DW MRI, and axial pre-contrast T1-weighted axial 3D fast field echo DCE MRI sequences. A prior study (26) described their detailed sequence parameters.

### Spatial registered hypercube assembly: Image processing, pre-analysis

DCE images consist of a time series at fixed locations in the prostate, encompassing the entire prostate. These images display the evolution in time of contrast material over several hundred seconds following injection. The DCE shows contrast uptake in the tissues. By analyzing the DCE and exploiting the unique tumor physiology, a portion of tumors may be identified. The tracer concentration in the tissue that supplies and empties through the tumor vasculature is described by a simple two compartment model (23, 44, 45). For times greater than the time to reach the peak uptake of the contrast material in a tumor (>50 s), every voxel was fitted with an exponentially decaying function to form the washout (k_ep_) images and the probability likelihood (prob) images.

All MRI images were digitally resized (23–28) to 1 mm resolution in the transverse direction. Using the known location of the patient’s position on the table, all slices were resized to 6 mm spacing and aligned using resampling. The Dynamic Contrast Enhancement images were treated as the reference for spatial registration. Due to the short time intervals between scan types (<20 min), small rigid adjustments (minor transverse translation) were applied to the structural, diffusion, and DCE images. A “cube” is composed of stacked individual slices that were scaled, translated, and resliced to be spatially registered at the pixel level. These “ three-dimensional” (two transverse directions plus a spectral dimension composed of MP-MRI sequences) cubes were “stitched” together into a narrow three-dimensional hypercube to depict the entire prostate and other tissues in the field of view of the MRI scan. This stitching, or mosaicking, follows the approach used in remote sensing, in which large areas are stitched together. Mosaicking or stitching cubes greatly increases the processing speed for handling high-dimensional data. The spectral content of the hypercube had seven components (23–28): T1 (pre-contrast), T1 (maximum contrast), T2, ADC, DWI-high B (B = 1,000 s/mm^2^), Washout or k_ep_ from DCE.

### Anomaly detector generator: Anomaly detector (RX)

An anomaly detector (29–31) examines and computes statistics, such as mean value and covariance matrix, that characterize a background (the normal prostate organ) and identifies targets (tumor, benign prostatic hyperplasia) by noting voxels that quantitatively depart from the background. In contrast, supervised target detection uses a target signature to help distinguish a target from the background. This study, like many that examine multi- and hyperspectral images, applies the RX (32) algorithm to detect anomalous voxels. The RX algorithm queries each voxel and computes the voxel value and background statistics for all components, specifically the covariance matrix and mean. A voxel’s RX value (32) is the voxel’s Mahalanobis distance (Euclidean distance in whitened space) from the background (normal prostate) mean. A voxel’s large RX value shows a large deviation from the background. The RX decision surface is a hypersphere with background residing inside a sphere and anomalies outside. The covariance matrix corrects and accounts for correlations among the different components (for example, the correlation between ADC and DWI) to get a true measure of the aggregate contribution of each component to the deviation of the voxel from the background. Actual data fails to follow the ideal RX probability distribution, namely a chi-square distribution (32) requiring *ad-hoc* anomaly cutoff thresholds or employing acceptable false alarm rates. The Appendix summarizes some of the mathematics behind the RX algorithm. For more details, see references (29–32).

### Anomaly detector generator: Filtering noise

Computing the RX covariance matrix generates principal components (46). Principal components are linear combinations of all MRI components but are orthogonal or totally decorrelated from each other. The principal components are ordered based on their eigenvalues or statistical variation. Well-resolved images have large eigenvalues and high variation. In contrast, noisy principal components have small eigenvalues. Noise is reduced by filtering and eliminating the noisy (low eigenvalue) principal components, resulting in a more accurate RX calculation. The Appendix summarizes some of the mathematics behind the filtering of principal components. For more details, see references (27, 28, 47).

### Anomaly detector generator: Regularization and shrinkage

Regularization is another way to correct for the imperfections of the computed covariance matrix. The statistics describing the background (normal prostate) should follow a normal distribution. However, the analytical formula for the covariance matrix results in only an approximation. The goal of shrinkage regularization (27, 28, 48) is to perturb the original covariance matrix CM(γ) by mixing in a diagonal matrix with a mixing parameter γ to generate a regularized or modified regularized covariance matrix. The appropriate γ is chosen to maximize the normal distribution. Regularized or modified regularized covariance matrix generation follows the same procedure but differs in the mixing diagonal matrix. The Appendix summarizes some of the mathematics behind regularization. For more details, see references (27, 28, 48).

### Anomaly detector generator: Elliptical volume minimization

Elliptical volume minimization (EVM) (49) provides another approach for reducing the effects of noise in the covariance matrix calculation. EVM does not use an analytical solution. Instead, EVM sequentially removes 10% of randomly chosen pixel searches and computes and records the hypervolume elliptical volume for the remaining 90% of the prostate pixels. The minimum elliptical volume after the search is chosen, presumably reducing the effects of the 10% aberrant voxels.

### Reference mask: Color quantification: CIELAB

Perceiving color is a neuro-psychological phenomenon that depends on the observer and display (50–52). Objectively quantifying color to assess images is, therefore, fraught with challenges. However, considerable empirical research and effort have allowed for the conversion of color perception into quantitative metrics, specifically by using the CIELAB color space, also referred to the as L*a*b* color space (50–52). CIELAB is designed to relate to the CIE standard observer. The CIE standard observer is generated from color matching experiments conducted under laboratory conditions. The CIELAB is designed to be independent of any device, such as a computer monitor or a printer. It is based on the opponent color model of human vision, where red and green form an opponent pair, and blue and yellow form an opponent pair. Color is described by three values: L* for perceptual lightness and a* and b* for the four unique colors of human vision: red, green, blue, and yellow. The L* defines black at 0 and white at 100. The a* axis follows the green–red opponent colors, with negative values toward green and positive values toward red. The b* axis represents blue–yellow opponents, with negative numbers toward blue and positive toward yellow. Yellow for this study is related to tumor in this study and is of most interest. The Appendix summarizes some of the mathematics behind coloring. For more details, see references (50–52).

### Reference mask: ACE

A target, such as a tumor, can be characterized by its spectral signature. The spectral signature is a vector whose components are values from each of the MRI modalities. The tumor signature differs from the background (normal prostate) vector. The difference between the tumor and normal prostate vectors is exploited by supervised target algorithms. The adaptive cosine estimator (ACE) is one example of supervised target detection. The Appendix summarizes some of the mathematics behind ACE. For more details, see references (22–28, 31).

### Assessment: Receiver operator characteristic

The receiver operator characteristic (ROC) curve (53) evaluates the performance of a binary target detection algorithm, specifically the RX anomaly detector and its variants (filtering, regularization). In this study, for a given RX anomaly detector threshold, each voxel in normal tissue is classified as either a target (above the RX threshold) or a normal prostate (below the RX threshold). This study uses two types of reference images, namely ACE (threshold = 0.65) and CIELAB (threshold = 0.35), to depict the targets. The ROC curve displays the sensitivity (how well RX characterizes targets) and the 1-Specificity (how well RX characterizes background) for all RX thresholds. The area under the curve (AUC) from the ROC curve and the Youden Index (YI) or maximum accuracy summarize RX performance. The Appendix summarizes some of the mathematics behind the ROC curve. For more details, see reference (53).

### Reference mask and assessment: Reference masks/threshold cutoffs

Ideally, a “ ground truth” image mask depicts the actual locations, sizes, and shapes of the tumors. Current practice attributes “ ground truth” to the pathologist’s assessment and markings on the histology slides derived from a whole-mount prostatectomy. Pathology evaluation is acceptable for Gleason score and tumor volume determinations. However, pathology assessment of the histology for tumor location and position for MRI suffers from a few limitations. The histology preparations can result in distortions, shrinkage, and tearing. Tissues imaged by MRI are supported by muscles and other soft tissues and subject to gravity, further complicating their registration to histology slides. Due to the absence of any registration points, it is impossible to precisely register the histology slides to the axial MRI images in both the axial and transverse directions.

The radiologist’s delineation of tumors on the multi-parametric MRI might have served as candidate “ground truth” but it was not available. Instead, the images derived from ACE and Color/CIELABS applied to the spatially registered MRI marked the tumors at the voxel level for the ROC curve computations. In-scene signatures were inserted into the ACE calculations. The thresholds from the ACE or Color/CIELABS were taken from a previous study (24) that computed the correlation coefficients of tumor volumes derived from the ACE and Color/CIELABS using varying thresholds with the tumor volume generated from the pathologist’s evaluation of the slides taken from wholemount prostatectomy. The highest correlation was achieved with 0.65 and 0.35 for ACE and color/CIELABS, respectively, and was therefore chosen for this study. In practice (31, 32), RX applied to data does not follow the expected Chi-Square distribution. In practice, *ad hoc* or acceptable false alarm rates set the cutoff threshold values.

## Results

The patient average (± standard error) for the AUC and YI for the ROC curves for RX for all 26 patients are shown in **Tables 1**, **2**, respectively. For both calculations, the covariance matrix was filtered by eliminating 3 and 4 principal components. This was followed by applying modified regularization and regularization, as well as searching for the minimum elliptical volume. In addition, RX was generated, and ROC curves were computed using an unprocessed covariance matrix. The best performance in terms of highest AUC and YI was from the filtered covariance matrix approach and from applying the regularization to the covariance matrix. Elliptical volume minimization performed even worse than using an unprocessed covariance matrix.

**Table 1:** Average Area Under the Curve (AUC) and [standard error].

	Delete three PC	Delete four PC	Modified Regularization	Regularization	Elliptical Envelope	Unprocessed
**ACE**	0.734[0.022]	0.727[0.022]	0.638[0.017]	0.716[0.017]	0.544[0.025]	0.581[0.018]
**CIELAB B**	0.742[0.025]	0.740[0.022]	0.643[0.023]	0.722[0.022]	0.508[0.024]	0.569[0.020]

The covariance matrix corrections are denoted as gray. ACE (threshold=0.65, denoted as blue) and CIELAB (threshold 0.35) are the Reference images.

**Table 2:** Youden Index and [Standard Error].

	Delete three PC	Delete four PC	Modified Regularization	Regularization	Elliptical Envelope	Unprocessed
**ACE**	0.706[0.017]	0.727[0.016]	0.639[0.012]	0.690[0.013]	0.597[0.017]	0.608[0.012]
**CIELAB B**	0.711[0.020]	0.708[0.018]	0.648[0.016]	0.695[0.018]	0.605[0.014]	0.615[0.012]

The covariance matrix corrections are denoted as gray. ACE (threshold=0.65, denoted as blue) and CIELAB (threshold 0.35) are the Reference images.

Unlike processing using ACE and CIELAB (24), anomaly detection failed to achieve high correlation with histology-derived tumor volume or those derived from manual coloring.

## Discussion

This is the first spatially registered multi-parametric MRI study to apply an unsupervised target detection algorithm, namely the RX algorithm for prostate cancer. The best performance in terms of highest AUC and YI were the filtered covariance matrix approach, and from applying regularization to the covariance matrix. Elliptical volume minimization performed even worse than using an unprocessed covariance matrix. The anomaly detection attained high AUC and YI from ROC when using ACE and CIELAB color images as reference images. However, unlike supervised target detection, anomaly detection failed to achieve high correlation with histology derived tumor volume or those derived from manual coloring.

Imaging is only one way to non-invasively evaluate a patient for the possible presence of prostate cancer. Recent research (54) evaluated biomarkers residing in urine or blood (beyond prostate serum antigen tests) to determine whether a patient has prostate cancer, its stage, and its potential aggressiveness. Using metrics derived from imaging does not preclude using biomarkers. Greater accuracy might be achieved by combining the new biomarkers with predictors derived from algorithms applied to spatially registered hypercubes. Each patient can be individually evaluated by determining the presence of biomarkers and computing imaging metrics to generate a patient-specific probability for the presence of prostate cancer and its likelihood to metastasize and extend beyond the prostate.

It is important to note that the “ reference image” for the prostate tumors in this ROC curve study for anomaly detection was taken from ACE and CIELAB images. Tumor delineation from pathologists ‘ histology images is available but is not a good reference for this study. The histology images suffer from distortion and shrinkage during the slicing, staining and preservation processes and are not subject to stresses from connections to muscles and other soft tissues as well as gravity. Using ACE and CIELAB images can also be problematic due to their unverified connection to pathology assessed histology slides. However, ACE and CIELAB are perfectly spatially registered to the RX detection images. The ACE and CIELAB images also describe classic tumor behavior, i.e., ones that exhibit low diffusion but high vasculature. Future investigation is warranted to use “ reference image” masks generated with multiple signatures for ACE and/or some green (low vasculature, low diffusion) CIELAB images.

Anomaly detection is sensitive to volumes within the prostate that do not necessarily display the spectral characteristics of an archetypal tumor, namely one that shows low diffusion but high vascularization. Malignant tumors can show limited vascularization but limited diffusion and, therefore, can be sufficiently spectrally anomalous to be detected by RX but not by supervised target detection. However, anomaly detectors may also detect hyperplasia, or swelling, within the prostate. In addition, anomaly detection is sensitive to image artifacts such as misregistration in multi-parametric MRI.

Previous work (27) on Signal to Clutter Ratio and Gleason score found that the covariance matrix was more optimally handled by deleting three principal components, not four principal components. Earlier work also found that a better-performing RX used a covariance matrix treated with a modified regularization procedure, not the more standard regularization procedure. In contrast, the more optimal covariance matrix for anomaly detectors was generated by filtering four principal components, not three. The standard regularization performed better than the modified regularization for generating a more optimal covariance matrix and likelihood and a better performing RX.

The large ROC AUC and large Youden Index relating the RX to the supervised target algorithm ACE and quantitative CIELAB coloring scheme suggest a strong relationship between anomalies and regions sharing typical tumor characteristics. However, the thresholds associated with the Youden Index vary considerably and unpredictably from patient to patient. ROC curves sample all classifier gray levels or thresholds. However, employ ing RX for tumor volume determination or prediction requires a single threshold. Previous studies and the present work find that RX do not obey expected the chi-squared distribution (31), complicating efforts to set detection thresholds. Employing RX to reliably predict tumor volume requires an appropriate threshold. Further work is needed to identify an appropriate RX threshold for determining tumor volume.

This study has some limitations. Future work should employ radiologist-delineated tumors on the MRI as the “ ground truth.” The threshold parameters for ACE, CIELABS, and ultimately RX should be checked through cross-validation studies. The patients in this study were prospectively enrolled but were analyzed retrospectively from a single institution (NIH). Clinical implementation variations, therefore, could not be examined, and the effects of variation on this analysis are uncertain. In addition, as with all retrospective analyses, the findings herein may be subject to biases. Lastly, the dataset comprised only 26 patients. Although a limited number of patients were assessed, consecutive patients were analyzed to minimize potential bias, and nevertheless highly statistically significant AUC and YI values were achieved, showing the potential clinical value of this approach.

## Data availability statement

The raw data supporting the conclusions of this article will be made available by the authors, without undue reservation.

## Ethics statement

Ethical review and approval were not required for the study on human participants in accordance with the local legislation and institutional requirements. Written informed consent for participation was not required for this study in accordance with the national legislation and the institutional requirements.

## Author contributions

Conception and design: RM. Administrative support: RM, CS, and PC. Provision of study materials or patients: BT and PC. Collection and assembly of data: RM, BT, and PC. Data analysis and interpretation: RM. All authors listed have made a substantial, direct, and intellectual contribution to the work and approved it for publication.
