# Supplementary material for: Assessing and testing anomaly detection for finding prostate cancer in spatially registered multi-parametric MRI
Source: Front Oncol. 2023 Jan 5;12:1033323. doi: 10.3389/fonc.2022.1033323 (PMC9869917; doi:10.3389/fonc.2022.1033323)
Supplement: Supplementary file 1 [file DataSheet_1.pdf]

Appendix

Anomaly Detector (RX)

Like all detectors, an anomaly detector **(29-31)** generates a decision surface that demarks targets from background. In the case of RX **(32)**, a popular anomaly detector for multi- and hyperspectral images, the decision surface is a hypersphere in the whitened space as shown in Figure 1, a two-dimensional figure. The anomaly detector, unlike supervised target detection, does not employ a target signature that characterizes the target or tumor. Instead, a target is defined as one that resides outside the background or normal prostate. The background resides inside the hypersphere while the targets reside outside. The scalar value for an anomaly detector RX(x_i) for voxel i and vector x_i (7 components in this study) is

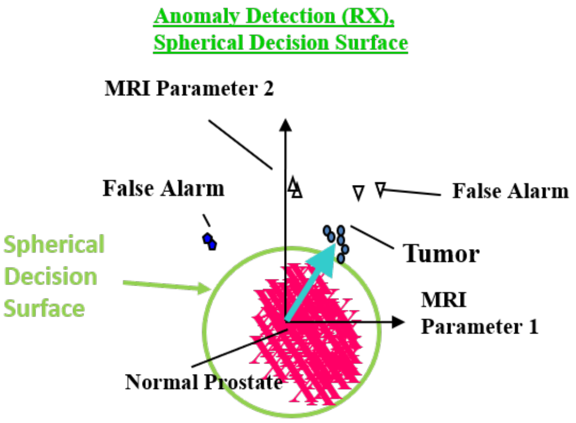

Figure 1 Two Dimensional Schematic picture of Anomaly Detection Decision Surface (hypersphere, shown as a circle in 2D) with Tumor, Targets and False Alarms outside circle, normal prostate or background inside circle

$$RX\ (x_i) = (x_i - \mu)^T CM^{-1}(x_i - \mu) \qquad (1)$$

Where μ is the background vector, CM is the multispectral covariance matrix. The superscript T is the matrix transverse operator and superscript -1 is the matrix inverse and Equation 1 depicts a matrix multiplication.

Filtering noise

The inverse covariance matrix CM^{-1} is a square symmetrical matrix and decomposes into three parts **(46),**

$$CM^{-1} = \Lambda^T \lambda^{-1} \Lambda \qquad (2)$$

namely the eigenmatrix Λ, transpose of the eigenmatrix Λ^T, and diagonal matrix λ^{-1} with eigenvalues $\lambda^2_1, \lambda^2_2, \lambda^2_3 \ldots \lambda^2_M$ populating the diagonal

$$\lambda^{-1} = \begin{bmatrix} \frac{1}{\lambda_1^2} & 0 & .... & 0 & 0 \\ 0 & \frac{1}{\lambda_2^2} & & & 0 \\ ... & & ... & & ... \\ 0 & & & \frac{1}{\lambda_{M-1}^2} & 0 \\ 0 & 0 & ... & 0 & \frac{1}{\lambda_M^2} \end{bmatrix} \qquad (3)$$

The eigenvalues are ordered according to size ranging from the largest λ_1 to the smallest λ_M. The images corresponding to the eigenvalues and eigenvectors range from high signal and variation (1,2) to low variation and very noisy (M-1, M). The lowest value eigenvalues (M-1, M) elevate the noise due to the inversion in the inverse matrix CM^{-1} (Eqs. [2,3]). Filtering out the noisy eigenvectors (**27,28,47)** means removing or deleting the lowest valued eigenvalues (3 or 4 in this study) from the inverse matrix i.e.,

$$\lambda_{Filtered}^{-1}{}^{-1} = \begin{bmatrix} \frac{1}{\lambda_1^2} & 0 & & 0 & 0 \\ 0 & \frac{1}{\lambda_2^2} & & & 0 \\ ... & & ... & & ... \\ 0 & & & 0 & 0 \\ 0 & 0 & ... & 0 & 0 \end{bmatrix} \qquad (4)$$

and inserting Eq. [4] into Eq. [2]

$$CM_{Filtered} = \Lambda^T \lambda_{Filtered}^{-1} \Lambda \qquad (5)$$

resulting in

$$RX_{Filtered}(x_i) = (x_i - \mu)^T CM_{Filtered}^{-1}(x_i - \mu) \qquad (6)$$

after inserting Eq. [5] into Eq. [1].

Regularization and shrinkage

The goal of shrinkage regularization (**27,28,48)** is to perturb the covariance matrix CM(γ) to maximize the normal distribution, or equivalently minimize the discriminant function d(γ) [=-ln(normal

distribution)] by adding a diagonal component that is controlled by the parameter γ and thereby perturbing the covariance matrix CM into the regularized $CM_{mod\_Reg}(\gamma)$

$$CM_{\mathrm{mod\_Re}g} = (1-\gamma)CM + \gamma V \qquad (7)$$

V is a diagonal matrix filled up with the square of the standard deviations from M modalities is given by

$$V = \begin{bmatrix} \sigma_1^2 & 0 & & 0 & 0 \\ 0 & \sigma_2^2 & & & 0 \\ \ldots & & \ldots. & & \ldots \\ 0 & & & \sigma_{M-1}^2 & 0 \\ 0 & 0 & \ldots. & 0 & \sigma_M^2 \end{bmatrix} \qquad (8)$$

Using Eqs. [7,8] the modified discriminant function d_{mod_Reg} (γ)

$$d_{\mathrm{mod\_Re}g}(\gamma) = \sum_{i=1}^{N} (x_i - \mu)^T CM^{-1}_{\mathrm{mod\_Re}g}(\gamma)(x_i - \mu) + \ln(\det(CM_{\mathrm{mod\_Re}g}(\gamma))) \qquad (9)$$

is computed for $0<\gamma<1$ and a minimum $d_{mod}(\gamma_{min})$ is found at $\gamma_{min.}$ resulting in a CM_{Mod_Reg} and RX_{Mod_Reg} (Eq. [10,11]) using a modified regularization procedure (using Eqs. [7-9]).

$$CM_{\mathrm{mod\_Re}g\_Min} = (1-\gamma_{\min})CM + \gamma_{\min} V \qquad (10)$$

$$RX_{\mathrm{mod\_Re}g}(x_i, \gamma = \gamma_{\min}) = (x_i - \mu)^T CM^{-1}_{\mathrm{mod\_Re}g\_Min}(x_i - \mu) \qquad (11)$$

Another approach for reducing noise is through a more standard form of regularization and shrinkage **(27,28,48).** That is shrinking the difference in the highest and lowest eigenvalues by adding a diagonal component that is controlled by the parameter γ and thereby perturbing the covariance matrix CM into the regularized $CM_{Reg}(\gamma)$

$$CM_{\mathrm{Re}g} = (1-\gamma)CM + \frac{Tra(CM)}{M}\gamma I \qquad (12)$$

where Tra denotes the trace operator and I is the identity matrix and γ ranges from γ=0.0 or no CM modification to γ=1.0 or CM is proportional to the identity matrix. The goal of regularization is to perturb the covariance matrix CM(γ) so as to maximize the normal distribution, or equivalently minimize the discriminant function d(γ) (=-ln(normal distribution)) i.e.

$$d_{\mathrm{Reg}}(\gamma)=\sum_{i=1}^{N}(x_i-\mu)^T CM_{\mathrm{Reg}}^{-1}(\gamma)(x_i-\mu)+\ln(\det(CM_{\mathrm{Reg}}(\gamma))) \qquad (13)$$

where the sum is over all N samples in the prostate ensemble and det denotes the determinant operation. A search is conducted among the γ's (range $0<\gamma<1$) for the lowest discriminant function that can be achieved with $\gamma_{\min}$ and results in a regularized RX_{Reg}

$$RX_{\mathrm{Reg}}(x_i,\gamma=\gamma_{\min})=(x_i-\mu)^T CM_{\mathrm{Reg\_Min}}^{-1}(\gamma=\gamma_{\min})(x_i-\mu) \qquad (14)$$

It should be noted that γ=0 results in the standard RX (Equation 1).

Color quantification: CIELAB

Color quantification proceeds in several steps. The parameters from the equations are derived form experiments.

First, three bands (of the seven bands) from the spatially registered hypercube **(23-28**) must be selected and assigned in order to highlight tumors. Tumors show elevated vascularization but lower diffusion. Therefore, the washout or Ktrans is assigned to Red, the DWI with highest gradient magnetic field High-B is assigned to Green, and the ADC is assigned to Blue. Tumors should yield high Red and Green but low Blue, resulting in tumors appearing as yellow.

Next, each voxel i in band q must be normalized $x_{norm,i,q}$ based on the maximum voxel value $x_{max,q}$ and minimum voxel value $x_{min,q}$ i.e.

$$x_{norm,i,q}=\frac{x_{i,q}-x_{min,q}}{x_{max,q}-x_{min,q}} \qquad (15)$$

To account for the non-linear perceptual differences based on intensity, the RGB normalized values where 0 denotes Red is denoted by q=0, Green is denoted by q=1 and Blue is denoted by q=2 and scaled

$$RGB(x_{norm,i,q})=100\left(\frac{x_{norm,i,q}+.055}{1.055}\right)^{2.4} \quad for\ x_{norm,i,q}>0.0455 \qquad (16a)$$

$$RGB(x_{norm,i,q})=100\left(\frac{x_{norm,i,q}}{12.92}\right) \quad for\ x_{norm,i,q}<0.0455 \qquad (16b)$$

Psychovisual studies account for wavelength overlap in the visual cones and human response to light intensity from different wavelengths, is expressed in the transformed XYZ coordinate system from the RGB system, assuming a standard Illuminant of D65

$$X(x_{norm,i}) = \left[RGB(x_{norm,i,0}) * 0.634 + RGB(x_{norm,i,1}) * 0.185 + RGB(x_{norm,i,2}) * 0.145\right] /95.047 \quad (17a)$$

$$Y(x_{norm,i}) = \left[RGB(x_{norm,i,0}) * 0.311 + RGB(x_{norm,i,1}) * 0.592 + RGB(x_{norm,i,2}) * 0.0974\right] /100.0 \quad (17b)$$

$$Z(x_{norm,i}) = \left[RGB(x_{norm,i,0}) * -.00119 + RGB(x_{norm,i,1}) * 0.00555 + RGB(x_{norm,i,2}) * 0.771\right] /108.88 \quad (17c)$$

To correct for the effects of illumination in each component X, Y,Z:

$$\overline{XYZ_\iota}(x_{norm,i}) = XYZ(x_{norm,i})^{.333}\ for\ XYZ(x_{norm,i})_i > 0.008856 \quad (18a)$$

$$\overline{XYZ_\iota}(x_{norm,i}) = 7.787 * XYZ_i\ \ (x_{norm,i}) + \frac{16}{116} \quad for\ XYZ(x_{norm,i}) < 0.008856 \quad (18b)$$

And finally, converting to the L*a*b* opponent color space,

$$L(x_{norm,i}) = 116 * \overline{Y_\iota}(x_{norm,i})\text{-}16 \quad (19a)$$

$$a(x_{norm,i}) = 500 * (\overline{X_\iota}\ \ (x_{norm,i}) - \overline{Y_\iota}\ \ (x_{norm,i})) \quad (19b)$$

$$b(x_{norm,i}) = 200 * (\overline{Y_\iota}(x_{norm,i}) - \overline{Z_\iota}(x_{norm,i})) \quad (19c)$$

Only the yellow component is relevant for computing the tumor in this application. Yellow component of the image is taken from the larger b-values. After thresholding, the b image provides the Reference Image mask for the tumor. Figure 2a shows a color slide from a representative patient (Patient #11) spatially registered hypercube, and from assigning red to Washout, green to the High-B DWI, and blue to the ADC. Figure 2b shows the mask from thresholding (=0.35) to the b portion of the CIELAB.

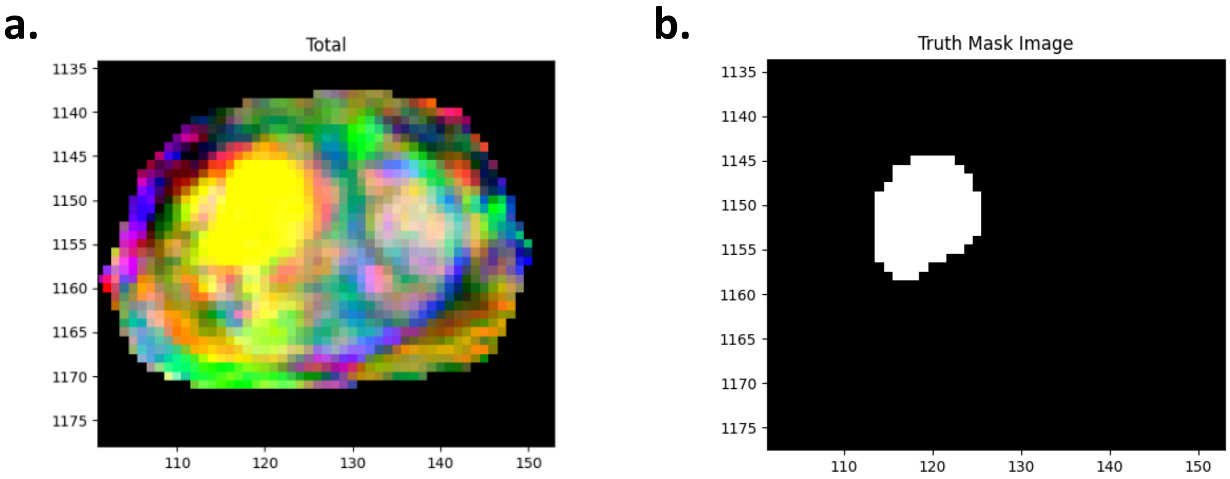

Figure 2a. Color display of a slice from Patient #11 by assigning red to Washout, green to DWI, High-B, blue to ADC. Figure 2b Shows the b portion of CIELAB with 0.35 threshold. Note the correspondence of yellow portion of Figure 2a and white in Figure 2b

ACE

The ACE (Eq. [20]) (**23-28, 31**) was transferred for this medical application (17-21) and applied to spatially registered MP-MRI. The algorithm (ACE) uses in-scene multispectral tumor signatures (for tumor). S is the target (tumor) signature and is a 7-component vector [DWI, T1, T2, ADC, DCE in this analysis (**23-28)]**. S, the in-scene tumor signature, is selected from yellow voxels in a three-color display of the spatially-registered MP-MRI (red is Washout, green is DWI, high-B, blue is ADC) (**23-28).** The component S_q (for vector S) is the average from T target vector-voxels $x_{p,q}$ summed over p target voxels (identified as yellow) (see Eq. [1]). μ is the background (normal prostate) 7 component vector. CM is the covariance (7×7) matrix for the background. The background voxels needed for m and CM were taken from digitally outlining the prostate on the spatially-registered MP-MRI (**23-28**).

$$S_q = \frac{1}{T}\sum_{p=1}^{T} x_{p.q}, \quad \mu_q = \frac{1}{N}\sum_{p=1}^{N} x_{p.q}, \; ACE(x_i) = \frac{(S-\mu)^T CM^{-1}(x_i-\mu)}{\sqrt{[(S-\mu)^T CM^{-1}(S-\mu)][(x_i-\mu)^T CM^{-1}(x_i-\mu)]}} \tag{20}$$

To compute the background statistics, the prostate image is manually outlined for all slices to generate an image mask and restrict computations to the prostate volume. ACE generates a conical hyperspace decision surface. A large angle (but small cosine, small ACE score, outside decision cone) or a small angle (but large cosine, large ACE score, inside the decision cone) determines whether a voxel is background (normal tissue) or target (tumor).

Receiver Operator Characteristic (ROC)

The Receiver Operator Characteristic curve summarizes **(53)** and helps assess a binary classifier by plotting the probability of target detection (or sensitivity) against the false alarm probability (or 1-specificity) for all threshold settings. As an example, the "Reference Image" (Figure 3a) for the tumor is derived from the threshold ACE (Figure 3a) or CIELAB image and is shown as white (binary value 1 from the mask image). In this study, the binary classifier (RX) identifies each voxel as tumor or normal prostate tissue. The classifier's (Figure 3b) accuracy is assessed by comparing the identified voxel with the Reference Image (Figure 3a). The entire prostate, that includes both the normal tissue and tumor (shown as white with a binary value of 1), is outlined and represented as a mask image (Figure 3c) and the black (or binary value 0) depicts areas that are not prostate. The Reference Image for the normal prostate is simply the difference in the Reference Image mask images between the entire prostate and the tumor image.

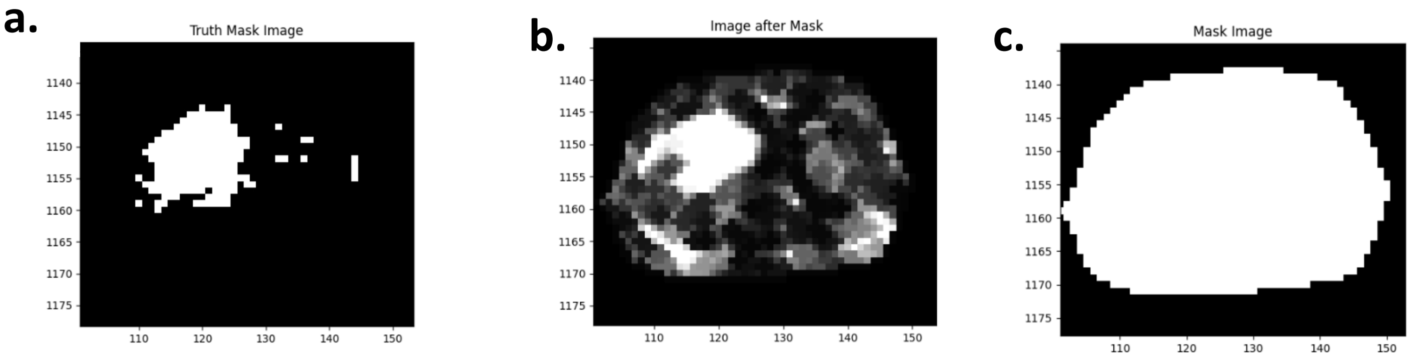

Figure 3a Mask from ACE, Threshold=0.65, Reference Image , Figure 3b, RX output, detection Figure 3c, Normal Prostate mask

The ROC vertical axis (Sensitivity) determines RX's ability to correctly evaluate the tumor voxels. The horizontal axis (False Alarm probability or 1-Specificity) assesses RX's assignment of normal prostate voxels. The RX image is a greyscale image with values ranging from a minimum (0) to a maximum value. From the

original RX image, mask images are generated based on dividing the RX range values into 100 discrete segments and polling each voxel whether it exceeded (or not) the divided RX range value. By polling all RX values, the ROC curve spans the lowest RX value (0) or 100% tumor detection and 100% False Alarm probability (upper right portion of the ROC curve) to the highest RX value or 0% tumor detection and 0% False Alarm probability. The optimal RX value, if feasible, would be 100% target detection and 0% False Alarm probability of the upper left corner for the ROC curve. The Area Under the Curve (AUC) is used to assess classifier. AUC ranges from 0 (poor performance) to 1 (optimal performance). In addition, another metric, the Youden Index (YI) scans RX values to find the combined maximum Sensitivity and Minimal False Alarm probability, i.e.

$$YI = \text{maximum}.5 * ((Sensitivity) + (1 - Specificity)) \tag{21}$$

YI ranges from 0 (poorest performance) to 1 (optimal performance).

Figures 4a, 4b show examples of ROC curves. Figure 5a displays ACE (threshold=0.65) as a Reference Image for prostate tumor and Figure 5b uses the CIELAB (threshold=0.35) to display yellow. The AUC is recorded and shown. The Youden Index is recorded and displayed as a green dot in the figures.

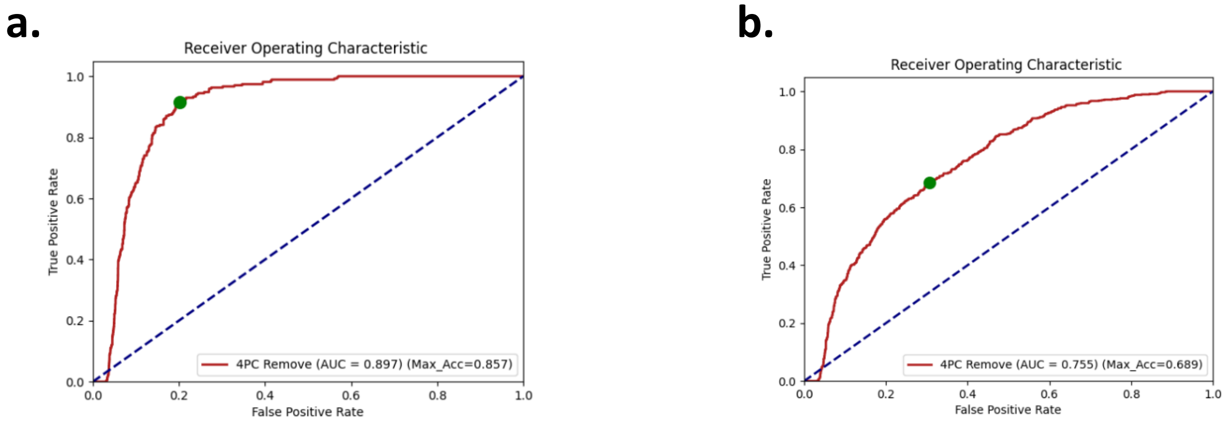

Figure 4a ROC curve for Reference Image ACE, Threshold=0.65, Green circle denotes the Youden Index point, Figure 4b ROC for Reference Image CIELAB, threshold=0.35, Green circle denotes the Youden Index.
